# T cell-NF-κB activation is required for tumor control *in vivo*

**DOI:** 10.1186/s40425-014-0045-x

**Published:** 2015-01-20

**Authors:** Sarah E Barnes, Ying Wang, Luqiu Chen, Luciana L Molinero, Thomas F Gajewski, Cesar Evaristo, Maria-Luisa Alegre

**Affiliations:** Department of Medicine, The University of Chicago, 924 E. 57th St. JFK-R312, Chicago, IL 60637 USA; Department of Pathology, The University of Chicago, 927 E. 57th St, Chicago, IL 60637 USA; Genentech, Inc., 1 DNA Way MS: 245c, South San Francisco, CA 94080 USA

**Keywords:** T cell, NF-κB, Tumor rejection, Priming, Effector function, Cytokine production, Cytotoxicity

## Abstract

**Background:**

T cells have the capacity to eliminate tumors but the signaling pathways by which they do so are incompletely understood. T cell priming requires activation of the transcription factors AP-1, NFAT and NF-κB downstream of the TCR, but whether activation of T cell-NF-κB *in vivo* is required for tumor control has not been addressed. In humans and mice with progressively growing tumors, the activity of T cell-intrinsic NF-κB is often reduced. However, it is not clear if this is causal for an inability to reject transformed cells, or if it is a consequence of tumor growth. T cell-NF-κB is important for T cell survival and effector differentiation and plays an important role in enabling T cells to reject cardiac and islet allografts, suggesting the possibility that it may also be required for tumor elimination. In this study, we tested whether normal T cell-NF-κB activation is necessary for the rejection of tumors whose growth is normally controlled by the immune system.

**Methods:**

Mice with genetically impaired T cell-NF-κB activity were subcutaneously injected with MC57-SIY tumor cells. Tumor growth was measured over time, and the anti-tumor immune response was evaluated using flow cytometry and cytokine detection assays.

**Results:**

Mice with impaired T cell-NF-κB activity were unable to reject tumors that were otherwise eliminated by wildtype mice, despite equal accumulation of tumor-reactive T cells. In addition, specific impairment of NF-κB signaling downstream of the TCR was sufficient to prevent tumor rejection. Tumor antigen-specific T cell-IFN-γ and TNF-α production, as well as cytotoxic ability, were all reduced in mice with impaired T cell-NF-κB, suggesting an important role for this transcription factor in the effector differentiation of tumor-specific effector T cells.

**Conclusions:**

Our results have identified the NF-κB pathway as an important signaling axis in T cells, required for the elimination of growing tumors *in vivo*. Maintaining or enhancing T cell-NF-κB activity may be a promising avenue for anti-tumor immunotherapy.

**Electronic supplementary material:**

The online version of this article (doi:10.1186/s40425-014-0045-x) contains supplementary material, which is available to authorized users.

## Background

T cells specific for tumor antigens can play an important role in the elimination of growing tumors [1]. However, the signaling pathways elicited in T cells following tumor antigen-dependent engagement of the TCR that are required for tumor rejection are not fully understood. TCR signaling includes the activation and nuclear translocation of several transcription factors, such as nuclear factor of activated T cells (NFAT), activator protein-1 (AP-1) and nuclear factor-κ-light chain enhancer of activated B cells (NF-κB) [2]. However, ablation of NFAT1 from T cells results in improved rather than impaired tumor control, underscoring the importance of NFAT1 in T cell anergy [3]. Nevertheless, while targeted disruption of NFAT1 or NFAT2 genes separately did not prevent T cell activation and IL-2 production, expression of a dominant negative NFAT in T cells did [4], suggesting that complete elimination of NFAT activity may prevent tumor control *in vivo*, although this has not been tested directly. Similarly, mice with genetic ablation selectively in T cells of c-Fos, an AP-1 subunit, displayed better, rather than worse, tumor control while mice with transgenic expression of c-Fos in T cells conversely experienced accelerated tumor growth [5]. In this case, the immunosuppressive effect of c-Fos was due to the induction in T cells of the negative regulator programmed death-1 (PD-1), a direct target of AP-1. As with NFAT, complete ablation of the AP-1 subunits might be expected to prevent tumor rejection. The functional role of the third main transcription factor of T cell activation, NF-κB, in tumor control remains to be determined.

Factors produced by tumors can directly or indirectly inhibit TCR-induced NF-κB activation, as shown with T cells isolated from patients with renal cell carcinoma [6], non-small cell lung cancer [7], or exposed to ascites fluid from an ovarian cancer patient [8]. Similarly, mice with growing tumors can harbor reduced NF-κB activity in peripheral T cells [9,10]. However, whether a reduction in T cell-NF-κB activity can be a cause for why T cells fail to control tumor growth and not just a consequence of tumor expansion, or whether other transcription factors can compensate *in vivo* for deficient NF-κB activity, remains to be tested. Understanding the signaling pathways that contribute to tumor rejection when it is successful may help design therapies to promote tumor elimination when it is not spontaneously achieved.

The transcription factor NF-κB comprises a family of proteins that include DNA binders (p50, p52) and DNA transactivators (RelA, RelB and c-Rel) [11]. In the absence of a stimulus, heterodimers of these subunits are retained in the cytoplasm by inhibitors of NF-κB (IκB). TCR activation results in the phosphorylation of the lipid raft-associated CAspase Recruitment domain Membrane-Associated guanylate kinase protein 1 (CARMA1) [12]. Phosphorylated CARMA1 associates with the protein B cell lymphoma 10 (Bcl-10), which acts as a scaffold for the mucosa-associated lymphoid tissue lymphoma translocation gene-1 (MALT1). The complex formed by CARMA1, Bcl-10, and MALT1 induces the activation of the IκB kinase complex IKK (IKKα, IKKβ and NEMO), which then phosphorylates IκB, an event that targets IκB for K48 ubiquitination and degradation by the 26S proteasome. This uncovers a nuclear localization domain within NF-κB dimers that enables them to translocate into the nucleus and initiate gene transcription.

Several genetic mouse models of NF-κB impairment in T cells have been generated, including the transgenic expression selectively in T cells of a mutated form of IκBα that cannot be degraded (IκBα∆N-Tg mice) [13], the conditional deletion of IKKβ (CD4-cre x IKKβ^fl/fl^ mice) [14] and the elimination of CARMA1 expression (CARMA1-KO mice) [15-17]. T cells from the first 2 strains have impaired NF-κB activation not only downstream of the TCR, but also of other receptors that activate NF-κB in T cells, such as tumor necrosis factor receptor (TNFR) family members and Toll-like receptor (TLR) family members. In contrast, TCR-dependent but not TNFR- or TLR-dependent NF-κB signaling is absent in CARMA1-KO T cells. Using these mice, our group and others have shown that T cell-NF-κB plays a role in the proliferation and survival of T cells. Because of its requirement in cell-cycle progression, T cell-NF-κB is important for Th1 and Th17 differentiation; however, if proliferation is rescued, Th1 differentiation can proceed whereas T cell-NF-κB controls Th17 differentiation at an additional downstream checkpoint, by enabling accessibility of the IL-17 locus [18-22]. Whereas T cell NF-κB is required for the thymic development of natural Tregs [23-27], and c-Rel can play a modest role in the differentiation of peripherally induced Tregs (iTregs) [25-27], T cell-NF-κB can antagonize iTreg differentiation when strongly induced at high antigen doses [28]. *In vivo*, the role of T cell-NF-κB has proven complex. For instance, its importance in the rejection of transplanted organs was dependent on the tissue origin of the graft. While IκBα∆N-Tg and CARMA1-KO mice failed to reject fully mismatched cardiac allografts and displayed delayed rejection of islet allografts, they successfully rejected skin allografts with only slightly delayed kinetics [21,29,30]. Given these pleiotropic and complex functions of T cell-NF-κB, its role in tumor control cannot be easily anticipated.

In the current study, we investigated whether normal activation of NF-κB in T cells is required for the rejection of tumors that are normally controlled by wildtype mice. Because IκBα∆N-Tg mice have defects in thymic selection resulting in reduced numbers of thymic and peripheral T cells perhaps due to the early expression of the transgene driven by the Lck promoter, CD4-cre x IKKβ^fl/fl^ and CARMA1-KO mice were employed to address this question, as they have closer to normal numbers of CD4^+^ and CD8^+^ conventional T cells. We found that mice with impaired T cell-NF-κB activity were unable to reject tumors that were otherwise eliminated by wildtype mice, and specific impairment of NF-κB signaling downstream of the TCR was sufficient to preclude anti-tumor responses. Reduced T cell-NF-κB activity did not reduce expansion of tumor-specific T cells, but resulted in decreased production of tumor antigen-specific IFN-γ and TNF-α, as well as diminished cytotoxicity against tumor antigen-expressing cells *in vivo*. Thus, T cell-NF-κB is required for the successful elimination of tumors. Our data identify this signaling pathway as a potential therapeutic target to enhance anti-tumor immunity in cancer patients.

## Results

### Rejection of MC57-SIY is dependent on T cell-IKKβ

**Figure 1:**
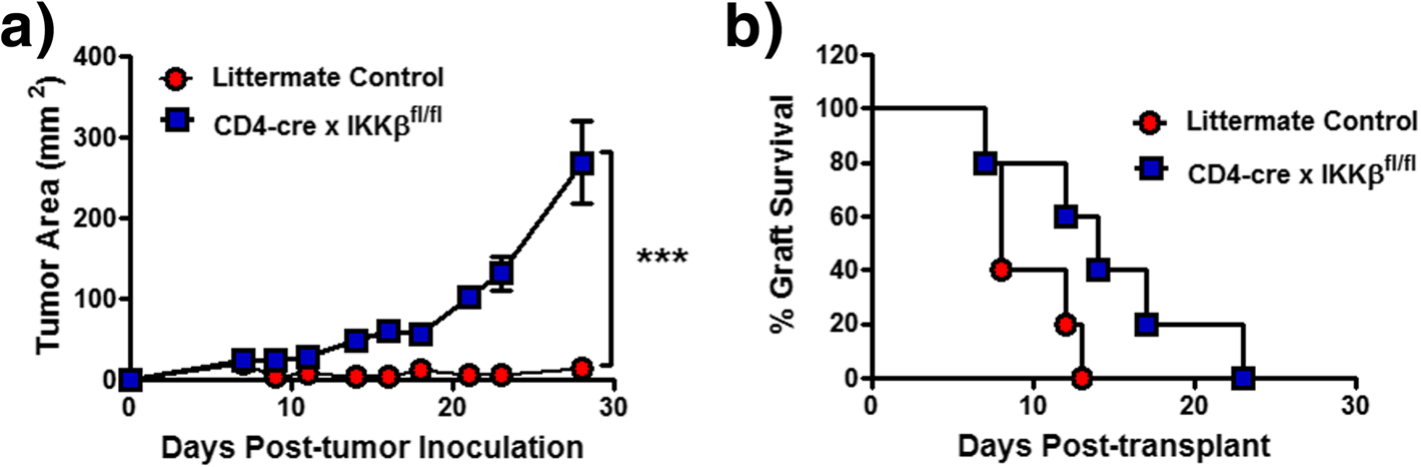
**IKKβ expression in T cells is required for MC57-SIY tumor rejection. a)** One million MC57-SIY tumor cells were subcutaneously injected into CD4-cre x IKKβ^fl/fl^ mice, and tumor growth was measured over time. **b)** Fully mismatched skin from wildtype BALB/c mice (H-2^d^) was transplanted into CD4-cre x IKKβ^fl/fl^ mice (H-2^b^). Results are representative of at least 2 experiments. ***p < 0.001.

To determine whether T cell-NF-κB signaling was required for tumor rejection, we examined the ability of T cell-NF-κB-impaired mice to reject a transplantable tumor. To this end, we utilized CD4-cre x IKKβ^fl/fl^ mice, the T cells from which lack the kinase IKKβ that is required for the phosphorylation of IκB and consequent nuclear translocation of NF-κB molecules. In these mice, cre-mediated deletion of IKKβ occurs at the double positive stage of thymocyte development, resulting in absence of IKKβ from all peripheral CD4^+^ and CD8^+^ αβT cells. CD4-cre x IKKβ^fl/fl^ mice have been previously described [14,31], and their T cells displayed normal survival and activation by polyclonal stimuli *in vitro*, expanded efficiently in response to superantigen administration *in vivo*, but were deficient in recall responses, help for germinal center formation, and lymphopenia-induced homeostatic proliferation [31,32]. CD4-cre x IKKβ^fl/fl^ mice and littermate controls (CD4-cre x IKKβ^+/fl^) were subcutaneously injected with 10^6^ MC57-SIY tumor cells, a mouse fibrosarcoma cell line engineered to express the model peptide antigen SIYRYYGL (SIY). This transplantable tumor is spontaneously rejected by wildtype mice [33]. While littermate controls successfully rejected the MC57-SIY tumors, CD4-cre x IKKβ^fl/fl^ mice did not (Figure 1a). To determine if this was due to an inability of CD4-cre x IKKβ^fl/fl^ mice to mount any kind of immune response when antigens were present in a cutaneous location, we examined whether they could reject skin grafts. CD4-cre x IKKβ^fl/fl^ mice (C57BL/6 background, H-2^b^) successfully rejected a fully mismatched BALB/c (H-2^d^) skin allograft, albeit with slower kinetics than wildtype mice (Figure 1b). Therefore, T cell-IKKβ was necessary for elimination of MC57-SIY tumors but was not required for the complete destruction of skin allografts.

### Initial T cell priming is unimpaired in CD4-cre x IKKβ^fl/fl^ mice

**Figure 2:**
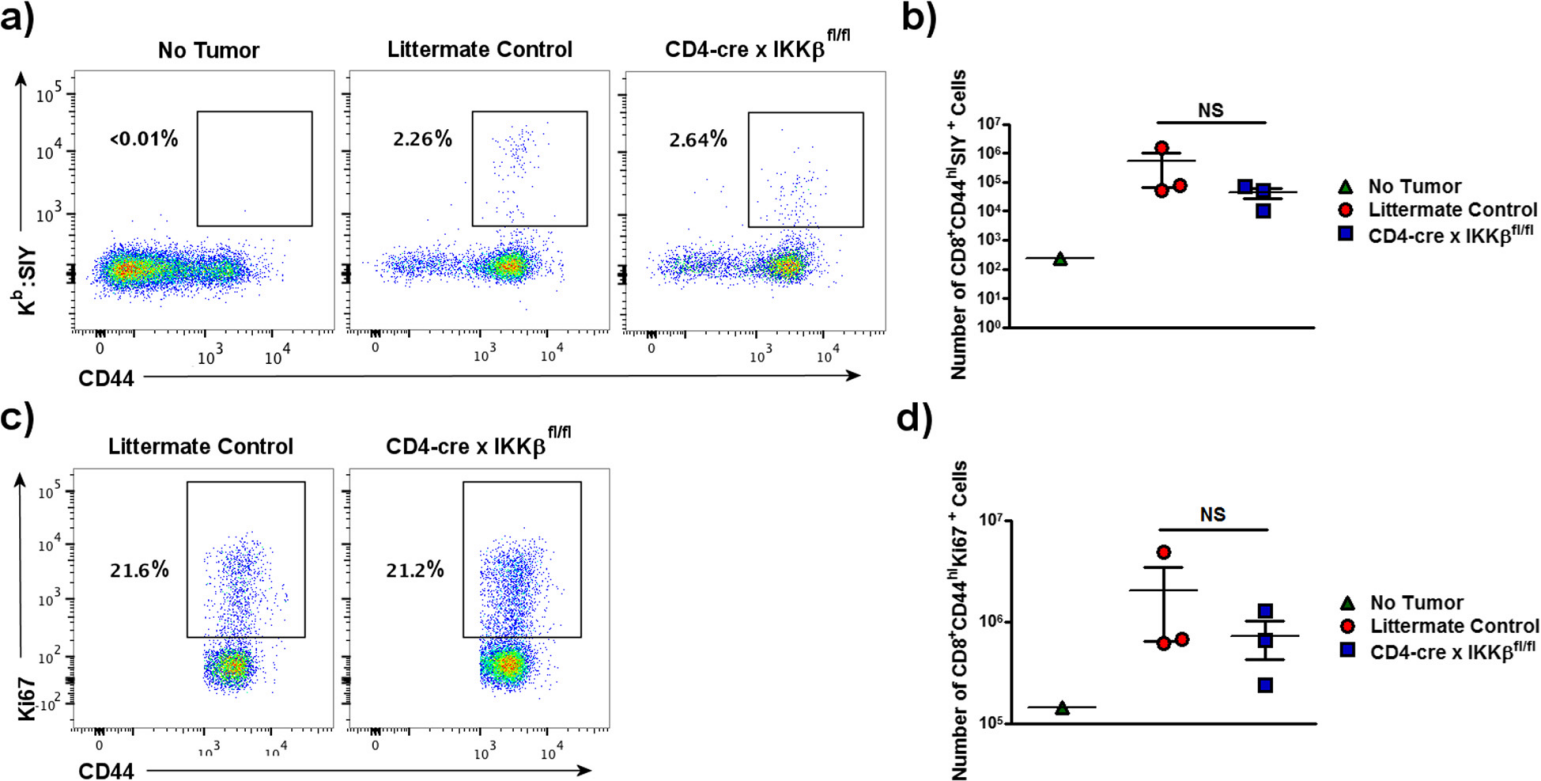
**Normal T cell priming in CD4-cre x IKKβ** ^**fl/fl**^ **mice.** CD4-cre x IKKβ^fl/fl^ mice were subcutaneously injected with 10^6^ MC57-SIY tumor cells, were sacrificed on day 7 and splenocytes were prepared for flow cytometry. Graphical representation **(a and c)** and absolute numbers **(b and d)** of SYI:Kb^+^ tumor-specific CD8^+^ T cells **(a and b)** and of Ki67^+^ proliferating CD8^+^CD44^hi^ cells **(c and d)**. Results are representative of at least 2 independent experiments. NS = not significant.

Lack of tumor rejection in CD4-cre x IKKβ^fl/fl^ mice may depend on impaired survival, proliferation, differentiation, migration or effector function of tumor-reactive T cells *in vivo*. To investigate the fate of anti-tumor T cells, we used fluorescently labeled pentamers of the MHC class I K^b^ molecule coupled to the SIY peptide to identify SIY-reactive T cells. Mice inoculated with MC57-SIY tumor cells were sacrificed on day 7, which was determined to be the height of the anti-tumor response [33], and splenocytes were analyzed by flow cytometry. Frequencies (Figure 2a) and absolute numbers (Figure 2b) of SIY-specific T cells were similar between CD4-cre x IKKβ^fl/fl^ mice and littermate controls, suggesting similar expansion and survival of SIY-specific CD8^+^ T cells following tumor exposure regardless of the presence or absence of IKKβ.

Because SIY is a model antigen, we also compared the proliferation of all activated CD8^+^ T cells that should also contain T cells reactive to non-SIY tumor antigens as well as tumor non-specific T cells. Splenocytes isolated 7 days following tumor inoculation were gated on CD8^+^CD44^hi^ populations and expression of Ki67, a nuclear protein induced during the active phases of the cell cycle, was assessed. As shown in Figures 2c and 2d, the proportion and total number of proliferating CD8^+^ T cells increased similarly following tumor injection in control and CD4-cre x IKKβ^fl/fl^ T cells, indicating that the initial T cell priming and cell survival following tumor implantation occurred normally despite lack of T cell-IKKβ.

### T cell-IKKβ is required for anti-tumor effector function

**Figure 3:**
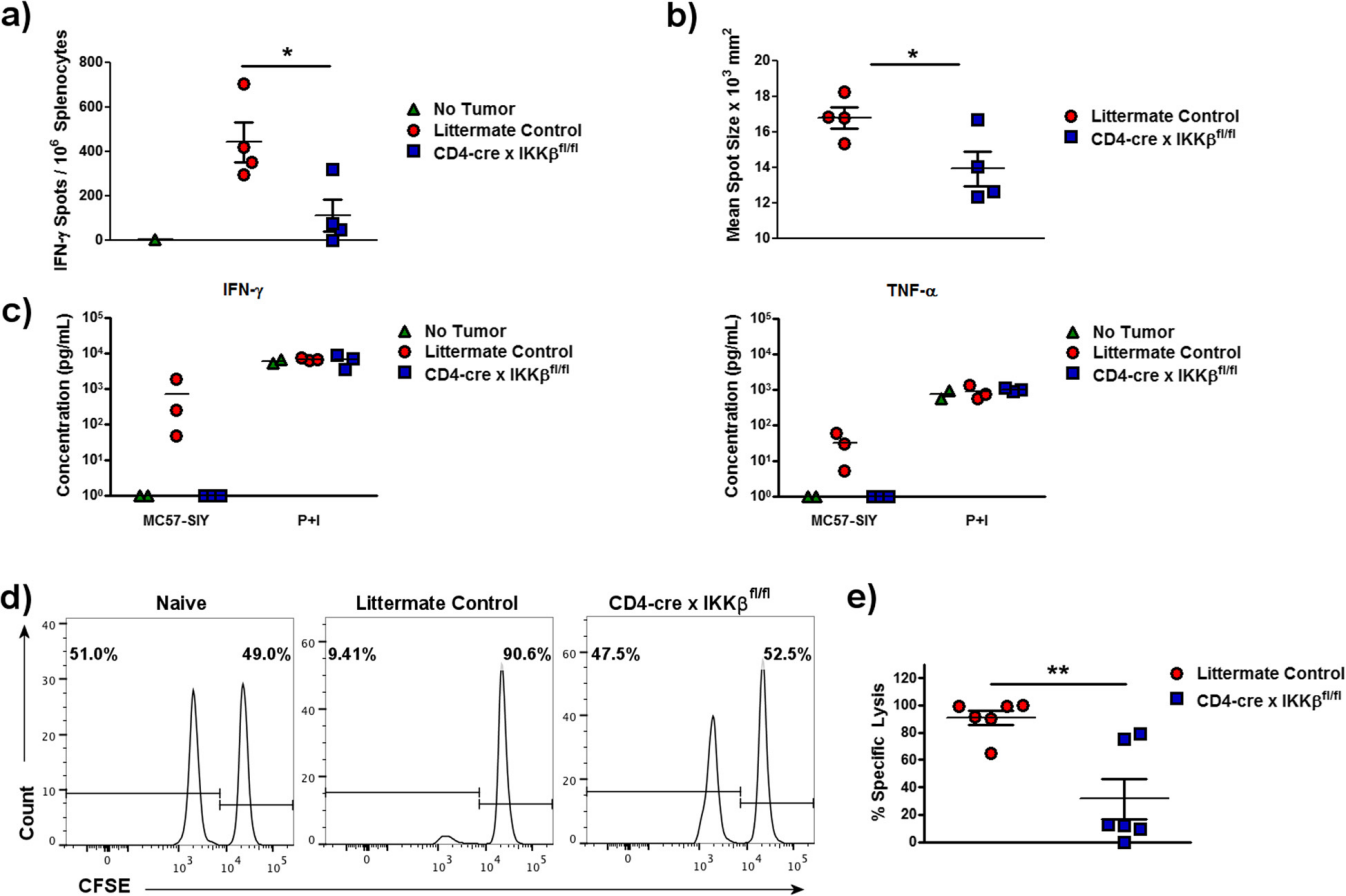
**T cell-IKKβ activity is required for anti-tumor effector function.** CD4-cre x IKKβ^fl/fl^ and littermate control mice were subcutaneously injected with 10^6^ MC57-SIY tumor cells and sacrificed 7 days later. **a)** Splenocytes were restimulated *in vitro* with γ-irradiated MC57-SIY tumor cells, and frequency of tumor-specific IFN-γ-secreting cells was determined by ELISpot. **b)** Mean spot size (from a) was used to evaluate amount of IFN-γ secretion on a per-cell basis. Results are representative of 2 experiments. **c)** Quantification of soluble IFN-γ and TNF-α from CD8^+^ splenocytes restimulated *in vitro* with γ-irradiated MC57-SIY tumor cells or PMA + ionomycin, as assessed by cytokine bead array. **d)** Mice bearing MC57-SIY tumors for 7 days were injected with a 1:1 ratio of CFSE-labeled cells loaded with (CFSE^low^) or without (CFSE^high^) SIY peptide. Eighteen hours later, mice were sacrificed and the presence of the target cells was assessed by flow cytometry. Results are representative of at least 2 experiments. **e)** Specific lysis of SIY-specific cells was calculated using the ratio of the transferred populations as described in Materials and Methods. Results combine 2 independent experiments. *p < 0.05, **p < 0.01.

To determine if a differentiation step downstream of T cell proliferation was affected by lack of IKKβ, IFN-γ production upon tumor antigen re-challenge *in vitro* was measured by ELISpot in splenocytes harvested 7 days post-tumor injection. Fewer CD4-cre x IKKβ^fl/fl^ than wildtype splenocytes secreted IFN-γ upon restimulation with irradiated MC57-SIY tumor cells (Figure 3a). Additionally, the production of IFN-γ from CD4-cre x IKKβ^fl/fl^ mice was reduced on a per-cell basis compared to littermate controls, as assessed by mean ELISpot size (Figure 3b).

Because cells other than T cells can produce IFN-γ, and to determine if CD8^+^ T cells specifically had impaired cytokine production in CD4-cre x IKKβ^fl/fl^ tumor-bearing mice, CD8^+^ T cells were enriched from the spleen on day 7 post-tumor implantation using magnetic bead-based negative selection, and restimulated with irradiated tumor cells or PMA + ionomycin. Cytokine secretion was assessed using a cytokine bead array. CD8^+^ T cells from CD4-cre x IKKβ^fl/fl^ mice had dramatically reduced IFN-γ and TNF-α production in response to tumor cell re-stimulation (Figure 3c). Surprisingly, these cells retained the ability to produce IFN-γ and TNF-α in response to PMA and ionomycin (Figure 3c and see Additional file 1: Figure S1), a combination of stimuli that bypasses the TCR and also stimulates the diversity of CD8^+^ T cells present in the culture. These findings suggest either that activated T cells were specifically unresponsive to tumor antigens upon re-challenge, or that they had failed to differentiate into IFN-γ- or TNF-α-producing cells in response to the tumor *in vivo*.

To further examine the extent of the functional deficiency in IKKβ-deficient tumor-specific T cells, we performed an *in vivo* cytotoxicity assay. CD4-cre x IKKβ^fl/fl^ mice and control littermates were injected with MC57-SIY tumor cells, and on day 7 post-inoculation, a 1:1 ratio of CFSE-labeled T cell-depleted splenocytes loaded with SIY tumor antigen (exposed to low CFSE concentration) versus left unloaded (exposed to high CFSE concentration) was transferred intravenously into each mouse. Mice were sacrificed 18 hours later and specific target lysis was calculated using the ratio of the two transferred populations evaluated by flow cytometry (Figure 3d). Compared to wildtype littermate controls, CD4-cre x IKKβ^fl/fl^ tumor-bearing mice demonstrated a very significant reduction in specific lysis (Figure 3e). Taken together, these findings suggest that, while other factors may contribute to the survival and proliferation of T cells, IKKβ expression is required to enable effector function during the anti-tumor immune response.

### Rejection of the MC57-SIY tumor is dependent on NF-κB signaling downstream of the T cell receptor

**Figure 4:**
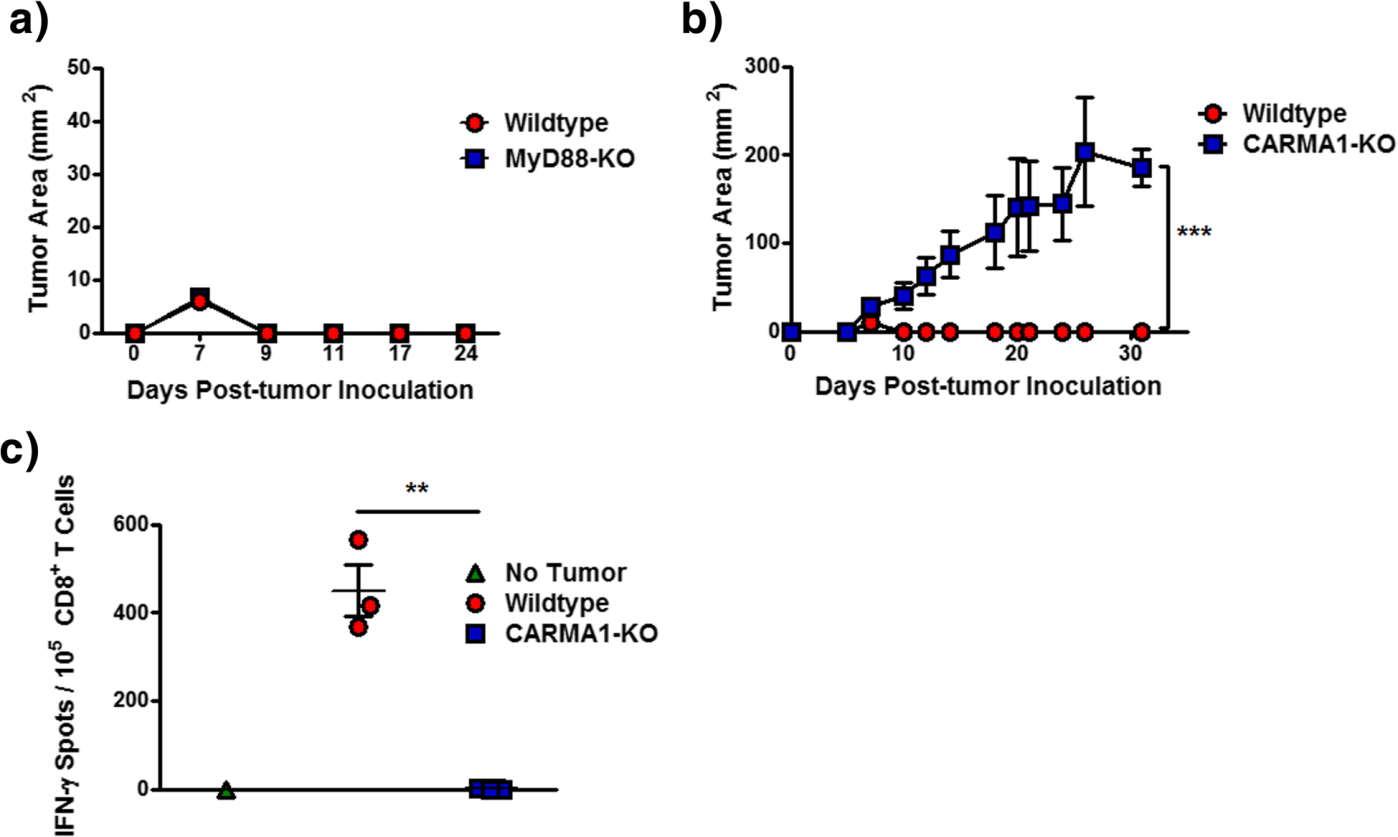
**Rejection of the MC57-SIY tumor is dependent on CARMA1 but not MyD88 expression.** One million MC57-SIY tumor cells were subcutaneously injected into either MyD88-KO **(a)** or CARMA1-KO **(b)** mice, and tumor growth was measured over time. **c)** Wildtype and CARMA1-KO mice were subcutaneously injected with 10^6^ MC57-SIY tumor cells and sacrificed 7 days later. Enriched splenic CD8^+^ T cells were restimulated with γ-irradiated MC57-SIY cells and an ELISpot was used to measure the frequency of cells secreting IFN-γ. The experiment was repeated 3 times with 3–5 mice per group/experiment. **p < 0.01, ***p < 0.001.

NF-κB in T cells can be activated by IKKβ downstream of several receptors, including TLRs, members of the TNFR superfamily, and the TCR. To assess whether T cell-IKKβ requirement for tumor rejection was due to TLR-dependent NF-κB activation as TLRs may detect damage-associated molecular patterns released during tumorigenesis, tumor rejection in MyD88-KO mice was analyzed. MyD88 is an adaptor downstream of most TLRs and necessary for TLRs to activate NF-κB [34]. MC57-SIY tumor cells were injected into MyD88-KO mice and wildtype controls both on the C57BL/6 background, and tumor growth was measured over time. Both MyD88-KO and wildtype mice rejected the tumors (Figure 4a), indicating that TLR-dependent NF-κB signaling is not required for the rejection of MC57-SIY tumors.

To address whether NF-κB signaling specifically downstream of the TCR was required for tumor rejection, tumor growth was analyzed in CARMA1-KO mice that lack the key adaptor connecting the TCR to the NF-κB activating machinery. Whereas wildtype mice successfully rejected the tumors, CARMA1-KO mice failed to prevent growth of MC57-SIY tumors (Figure 4b). As observed for CD4-cre x IKKβ^fl/fl^ mice, this was not due to a complete failure of CARMA1-KO mice to mount any immune response *in vivo*, as we have previously shown that they successfully reject fully allogeneic BALB/c skin grafts [21]. Similarly to CD4-cre x IKKβ^fl/fl^ mice, enriched CD8^+^ splenic T cells from tumor-bearing CARMA1-KO mice failed to produce IFN-γ in response to re-stimulation with irradiated tumor cells (Figure 4c), indicating a failure to mount a successful anti-tumor effector response. Taken together, these findings indicate that NF-κB activation downstream of the TCR is required for the elimination of MC57-SIY tumors.

## Discussion

NF-κB is activated in many cancer types and has also been associated with angiogenesis, tumor progression and metastasis [35]. For instance, we have shown that the development of acute T cell lymphoplastic leukemia driven by Notch-1 in mice depends on the ability of Notch to activate the NF-κB pathway [36]. Recently, NF-κB in parenchymal tumor cells was also shown to promote T cell recruitment in mice and humans [37]. However, the role of NF-κB within non-transformed T cells in their ability to control growth of parenchymal cancers had not been previously investigated. Our data show that both CD4-cre x IKKβ^fl/fl^ and CARMA1-KO mice fail to reject tumors that are otherwise eliminated by wildtype mice. Although the results point to the importance of T cell-NF-κB in tumor rejection, we cannot rule out that other signaling pathways downstream of IKKβ or CARMA1 may play a role in addition to, or instead of, NF-κB. Indeed, IKKβ has been shown to phosphorylate other substrates than IκB, including the Forkhead transcription factor FOXO3a, the mRNA destabilizer 14-3-3b, the insulin receptor substrate 1 and the docking protein 1 [38]. Similarly, CARMA1 has been shown to activate JNK2 in addition to NF-κB [39]. However, preliminary results indicate that IκBα∆N-Tg mice, in which T cells express a transgenic super-repressor form of IκBα, also have a defect in controlling MC57-SIY tumor growth (data not shown). The convergence of this same phenotype in 3 different genetic models, the common intersecting point being T cell-NF-κB impairment, strongly supports the hypothesis that normal activation of NF-κB in T cells is essential for tumor control *in vivo*. Moreover, other signaling pathways downstream of the TCR, such as NFAT or AP-1, cannot compensate for the absence of T cell-NF-κB. The tumor-controlling role of T cell-NF-κB suggests that augmenting the signaling of NF-κB may facilitate tumor rejection.

Although T cell-NF-κB has been implicated by us and by others in the survival and proliferation of conventional T cells in other model systems, our current results indicate that tumor-specific CD4-cre x IKKβ^fl/fl^ CD8^+^ T cells were able to expand and accumulate in tumor-bearing mice. Thus, other signaling pathways may be sufficient to support T cell proliferation and expansion in the absence of IKKβ *in vivo*. For instance, IKKα and IKKβ can substitute for one another for thymocyte development [40]. In addition, the lack of a defect in IKKβ-deficient T cell survival and proliferation in this tumor model allowed us to probe the role of NF-κB downstream of these early T cell priming events and identify an essential role for IKKβ in the acquisition of IFN-γ and TNF-α production by tumor-specific CD8^+^ T cells as well as of cytotoxicity *in vivo*. This observation is consistent with reports indicating that an NF-κB-binding site controls granzyme B transcription and that the c-Rel subunit of NF-κB (but not the p50 subunit) can bind to enhancer sites on the IFN-γ gene [41,42]. This impaired effector response is likely one of the mechanisms by which CD4-cre x IKKβ^fl/fl^ mice fail to reject MC57-SIY tumors, as T cell-IFN-γ and cytotoxicity are functions known to play important roles in the rejection of various growing tumors [43-45]. The fact that CD8^+^ T cells from CD4-cre x IKKβ^fl/fl^ tumor-bearing mice could produce IFN-γ in response to PMA/ionomycin suggests that tumor-specific IKKβ-deficient T cells may be anergic or hyporesponsive despite their initial proliferation, as anergic T cells have defects in Ras signaling which can be functionally rescued by bypassing early steps of T cell activation [46]. Alternatively, PMA/ionomycin may be eliciting IFN-γ from previously differentiated non tumor-reactive T cells, suggesting that IKKβ may be dispensable for effector differentiation in certain settings. It is conceivable that exposure of mice prior to tumor inoculation to environmental antigens in the presence of particular signals such as from pattern-recognition receptors, costimulatory molecules, or inflammatory cytokines may have enabled differentiation of a subset of T cells in the absence of IKKβ. Of note, while the expansion of CD4-cre x IKKβ^fl/fl^ CD8^+^ T cells was preserved in our studies, these cells displayed reduced binding to K^b^:SIY fluorescent pentamers (see Figure 2a), perhaps reflecting loss of high avidity clones. Indeed, varying TCR:ligand affinity in TCRβ mutant mice has been correlated with differential NF-κB activation and distinct CD8^+^ T cell fate decisions [47]. Thus, tumor immune responses in mice with impaired T cell-NF-κB may be limited because of deletion or lack of expansion of clones with high affinity for tumor antigens.

T cell-NF-κB is differently required for the elimination of distinct tissues *in vivo*, as it is essential for the rejection of cardiac but not skin allografts, although the tempo of skin rejection is slower than in control mice [29,48]. The fate of MC57-SIY tumors appears to segregate with that of cardiac rather than skin allografts, despite the tumors being positioned in a similar location as the skin graft bed. The underlying mechanisms for the differential role of T cell-NF-κB in the rejection of the various tissues are incompletely understood, but several factors likely play a role in providing sufficient signal strength to enable T cell activation despite reduced NF-κB. These may include differences in antigen load, site and quality of initial T cell priming and types of APCs present in the different tissues. For instance, we have previously shown that skin Langerhans cells but not splenic dendritic cells can activate NF-κB-impaired T cells and that transfer of donor-matched Langerhans cells is sufficient to drive rejection of cardiac allografts in T cell-NF-κB-impaired mice [49]. Whether absence of tumor control in mice with impaired T cell-NF-κB is due to these tumors lacking potent immune-stimulatory APCs remains to be demonstrated, but is consistent with the reduced T cell effector function observed in these mice.

NF-κB in T cells can be activated downstream of several surface receptors, including the TCR, most TLRs, and all TNFR family members [50]. The fact that CARMA1-KO but not MyD88-KO mice fail to reject MC57-SIY tumors suggest that TCR-NF-κB but not TLR-NF-κB plays a role in tumor control. TNFR family members are mostly expressed on activated rather than naïve T cells and include HVEM, CD27, CD30, OX40 and 4-1BB [51]. Engagement of these receptors by their respective soluble or membrane-bound ligands serves to costimulate activated T cells and results in TRAF-dependent activation of NF-κB [51-53], which is prevented by lack of expression of IKKβ but not of CARMA1. Whether NF-κB activity downstream of TNFR family members also plays a role in T cell-mediated spontaneous control of tumor growth remains to be investigated. Agonistic antibodies to 4-1BB, OX40 and CD27 that are immune-stimulatory are being tested as potential immunotherapies for cancer patients and the efficacy of anti-4-1BB pre-clinically has been shown to depend on TRAF2-mediated NF-κB activation [54].

Our data suggest that T cell-NF-κB is essential for the spontaneous rejection of transplantable tumors. It remains to be tested if it also plays a role in tumor surveillance and elimination of nascent autochthonous tumors. CD4-cre x IKKβ^fl/fl^ or CARMA1-KO mice do not appear to develop spontaneous tumors as they age, but this may be because the animals are kept in specific pathogen-free facilities and are not exposed to oncogeneic viruses or environmental carcinogens which may be necessary to drive tumor initiation [55]. Whether inhibition of T cell-NF-κB accelerates the development of spontaneous genetic or carcinogen-induced tumors, or prevents the efficacy of current immunotherapies such as blockade of the CTLA-4/B7 or PD-1/PDL1 pathways will be of interest to study in the future. In particular, we have previously shown that CTLA-4 and PD-1 inhibit TCR-mediated NF-κB activity [56,57] such that their blockade *in vivo* may conceivably function through enhancement of NF-κB signaling.

## Conclusions

T cell-NF-κB plays an important role in tumor control, indicating that reduced T cell-NF-κB observed in tumor-bearing hosts can be a cause of tumor progression and that other transcription factors cannot compensate *in vivo* for deficient NF-κB activity in T cells. These data suggest that enhancing this axis either genetically or with immunotherapies such as agonistic antibodies against TNFR family members are important therapeutic considerations. Based on our findings, we propose that the maintenance of NF-κB activity in T cells could facilitate tumor rejection by supporting T cell effector function, specifically pro-inflammatory cytokine secretion and specific cytotoxicity. It will be important to design such therapies to target only T cells, as enhancing NF-κB in cancer cells could contribute to tumor progression.

## Methods

### Animal models

C57BL/6 and BALB/c mice were obtained from Harlan Laboratories (Indianapolis, IN). CD4-cre mice, purchased from Jackson Laboratories [017336; B6.Cg-Tg(Cd4-cre)1Cwi/BfluJ], express the cre recombinase under the control of the CD4 promoter and have been previously described [58,59]. IKKβ^fl/fl^ mice, which express a *loxP*-flanked IKKβ locus and have been previously described [60], were generously provided by Dr. Michael Karin (University of California San Diego, San Diego, CA). CD4-cre mice were crossed with IKKβ^fl/fl^ mice in house (CD4-cre x IKKβ^fl/fl^ mice). CARMA1-KO mice were originally generated on the 129 background [16], were a gift from Dr. Daniel Littman (New York University, New York, NY) and were backcrossed for 6–10 generations onto the C57BL/6 background at the University of Chicago specific pathogen-free (SPF) barrier facility. MyD88-KO mice are deficient in the adaptor molecule MyD88 in all tissues [61] and were a gift from Dr. Alexander Chervonsky (University of Chicago, Chicago, IL). All mice were on the C57BL/6 background and bred at the University of Chicago SPF facility in agreement with our Institutional Animal Care and Use Committee (IACUC) and according to the National Institutes of Health guidelines for animal use.

### Tumor cell lines

The MC57 methylcholanthrene-induced fibrosarcoma cell line was provided by Dr. Hans Schreiber (University of Chicago, Chicago, IL). MC57-SIY was engineered to express the model antigen SIYRYYGL, which can be recognized by CD8^+^ T cells in the context of H2-K^b^ [33].

### Tumor challenge and measurement

Tumor cells were washed and resuspended in PBS at a concentration of 10^7^ cells/mL. A volume of 0.1 mL (10^6^ tumor cells) was injected subcutaneously into the right flank of each mouse. Tumors were measured with calipers by a single investigator, and tumor size was calculated as the product of the greatest tumor diameter length and its perpendicular width.

### Skin transplantation

The tail skin of euthanized BALB/c (H-2^d^) donor mice was cleaned with 70% ethanol. 1×1 cm segments of skin were removed with sterile scissors and attached onto a Petri dish maintained at 4°C, where they were kept moist with a sterile gauze soaked in 0.9% NaCl. C57BL/6 (H-2^b^) recipient mice were anesthetized with ketamine/xylazine. The surgical site was shaved and disinfected with povidone-iodine. Using sterile curved scissors, an area of flank skin was removed. The donor skin graft was placed onto the prepared graft bed, and secured with two Bandaids. After 7 days, the adhesive Bandaids were removed. Graft survival was assessed daily and rejection was defined as greater than 80% of the skin becoming necrotic.

### Splenocyte isolation

Spleens were homogenized in hypotonic ammonium chloride potassium (ACK) lysis buffer for 5 minutes to lyse red blood cells. Cells were resuspended in Dulbecco’s Modified Eagle Medium (DMEM, Invitrogen) supplemented with 5% fetal bovine serum (FBS; HyClone, Logan, UT), penicillin (100 U/mL), streptomycin (100 μg/mL), HEPES buffer (50 μM), β-mercaptoethanol (50 μM), and non-essential amino acids (1% final volume) (complete DMEM). Cells were filtered through cell strainers, centrifuged, and resuspended in complete DMEM. Live cells were counted with the Countess Automated Cell Counter (Invitrogen), using the trypan blue exclusion method.

### T cell enrichment

CD8^+^ T cells were enriched by negative selection using magnetic beads according to the instructions of the manufacturer (Stem Cell, Vancouver, Canada). Purity of T cells was verified in each experiment to be equal or superior to 85%.

### Flow cytometry and pentamer staining

Flow cytometric analyses were performed on single-cell suspensions stained in FACS buffer (PBS, 1% BSA, and 0.01% NaN_3_). Cells were then washed, resuspended in FACS buffer, and immediately analyzed by flow cytometry. Biotinylated pentamers of K^b^ complexed to SIY peptide (ProImmune, Oxford, U.K.) were used according to the manufacturer’s protocol and coupled to streptavidin-PE. Cells were labeled with PE-, PE-Cy7-, APC-, APC-Cy7-, or FITC- conjugated antibodies. The antibodies used targeted murine CD4, CD8α or CD8β, B220, or CD44. These antibodies were obtained from BD Biosciences (San Jose, CA), eBioscience (San Diego, CA), or Biolegend (San Diego, CA). In certain experiments, cells were stained with 7-AAD obtained from Invitrogen (Carlsbad, CA). For proliferation assays, cells were permeabilized, fixed and stained with APC-conjugated Ki67 according to the manufacturer’s instructions (BD Biosciences). Samples were acquired using Accuri, FACSCanto, or LSR Fortessa (BD Biosciences) flow cytometers. Data were analyzed using FlowJo software (TreeStar).

### ELISpot assay

The mouse IFN-γELISpot assay was conducted using the BD Bioscience (San Jose, CA) kit according to the manufacturer’s protocol. ELISpot plates were coated with anti-mouse IFN-γ antibody and stored overnight at 4°C. Plates were then washed and blocked with DMEM supplemented with 10% FBS for 2 hours at room temperature. Splenocytes (10^6^ cells/well) or enriched CD8^+^ T cells (2 × 10^5^ cells/well) were plated. Stimulation was performed with irradiated MC57-SIY tumor cells (20,000 rad) or PMA (50 ng/ml) and ionomycin (0.5 μg/ml) as a positive control. Plates were stored at 37°C in a 7.5% CO_2_ incubator overnight, washed, and coated with detection antibody for 2 hours at room temperature. They were again washed and coated with avidin-peroxidase for 1 hour at room temperature. Plates were washed and developed by addition of 3-amino-9-ethylcarbazole (AEC) substrate. Developed plates were dried overnight, read using an ImmunoSpot Series 3 Analyzer, and analyzed with ImmunoSpot software.

### Cytokine bead array

The mouse cytokine bead array was conducted using the Bio-Rad Laboratories Bio-Plex Pro™ Mouse Cytokine Th17 Panel A 6-Plex Group (Hercules, CA) kit according to the manufacturer’s protocol. Mice were challenged for 7 days with 10^6^ MC57-SIY tumor cells. CD8^+^ T cells were then isolated and restimulated with irradiated tumor cells or PMA + ionomycin. Supernatants were collected, and cytokine concentration was assessed. In addition, supernatants from PMA + ionomycin-stimulated samples were serially diluted and analyzed for IFN-γ content by ELISA to ensure that oversaturation of the assay was not masking differences between the samples.

### Cytotoxicity assay

Naïve syngeneic mice were sacrificed, and single-cell suspensions of whole splenocytes were prepared. T cells were depleted from splenocytes by negative selection for CD4^+^ and CD8^+^ cells using magnetic beads according to the instructions of the manufacturer (Stem Cell, Vancouver, Canada). Half of the remaining splenocytes were incubated with SIY peptide at a concentration of 1 μM at 37°C for one hour. All cells were washed. Peptide-loaded cells were stained with 0.5 μM CFSE, and remaining splenocytes were stained with 5 μM CFSE. Cells were counted using the Accuri flow cytometer, and equal populations of cells were intravenously transferred into tumor-bearing mice one week post-tumor inoculation. The day after transfer, tumor-bearing mice were sacrificed, and populations of transferred cells were analyzed by flow cytometry. Specific lysis was calculated using the formula, $$ \%\ \mathrm{Specific}\ \mathrm{Lysis}=\left[\raisebox{1ex}{${r}_{\mathrm{experimental}}$}\!\left/ \!\raisebox{-1ex}{${r}_{\mathrm{naive}}$}\right.\right]\times 100 $$ where $$ r = \frac{\%\ CFS{E}^{lo}\  cells}{\%\ CFS{E}^{hi}\  cells} $$.

### Statistical analyses

Comparisons of means were performed with GraphPad Prism (GraphPad Software) using the two-way ANOVA where appropriate with Bonferroni’s correction for multiple comparisons. Where appropriate, comparisons of means were performed with InStat software using the *t*-test. Differences were considered significant for p values <0.05.

## Additional file

Additional file 1: Figure S1. Stimulation with PMA + ionomycin elicits similar IFN-γ production from control and IKKβ-deficient CD8^+^ T cells. Supernatants from Figure 3c were serially diluted and the concentration of IFN-γ was measured by ELISA.
