# Supplementary figures and images for: T cell-NF-κB activation is required for tumor control in vivo
Source: J Immunother Cancer. 2015 Jan 20;3:1. doi: 10.1186/s40425-014-0045-x (PMC4308877; doi:10.1186/s40425-014-0045-x)

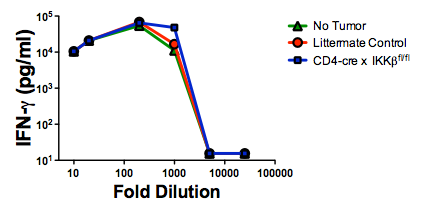

Supplement: Additional file 1: Figure S1. — Stimulation with PMA + ionomycin elicits similar IFN-γ production from control and IKKβ-deficient CD8+ T cells. Supernatants from Figure 3c were serially diluted and the concentration of IFN-γ was measured by ELISA. [file 40425_2014_45_MOESM1_ESM.png]
